# Ethnobotany of an indigenous tree *Piliostigma thonningii* (Schumach.) Milne-Redh. (Fabaceae) in the arid and semi-arid areas of South Omo Zone, southern Ethiopia

**DOI:** 10.1186/s13002-021-00469-6

**Published:** 2021-07-17

**Authors:** Mesfin Belete Hailemariam, Zerihun Woldu, Zemede Asfaw, Ermias Lulekal

**Affiliations:** 1 Department of Biology, Wolkite University, Wolkite, Ethiopia; 2 Department of Plant Biology and Biodiversity Management, Addis Ababa University, Addis Ababa, Ethiopia

**Keywords:** Benna-Tsemay, Consumption, Ethnic group, Hamer, Indigenous knowledge, Multiple uses, *Piliostigma thonningii*, South Omo Zone

## Abstract

**Background:**

Trees are important components of terrestrial ecosystems; they provide ecological, economic, and cultural services to humans. There is an urgent need for undertaking ethnobotanical investigations and documentation on the indigenous botanical knowledge of the local communities of a given area. This study was conducted to assess and document the categoric ethnobotany of *Piliostigma thonningii* and the associated indigenous knowledge of the local people related to use, management practices, and the threatening factors in the South Omo Zone of southwestern Ethiopia.

**Materials and methods:**

Six Kebeles were purposively selected from two districts of the zone and a total of 84 respondents were sampled, with consideration of gender, age, and wealth status. Data were collected using structured and semi-structured interviews, field observations, and group discussions. Relevant descriptive statistical methods were used to analyze the data. Ethnobotanical knowledge held by informants were computed using Pearson’s chi-square test and direct matrix ranking and pair-wise ranking was used to prioritize the uses according to community preferences and the level of the destructiveness of the reported threats.

**Results:**

The results showed that *P. thonningii* provides different functions to people’s livelihoods as shade, food, fodder, soil fertility, fuelwood, medicine, rope, multipurpose materials (e.g., Borketa for sitting and head support), huts, beehives, farm implements, chairs, fences, and timber. The indigenous knowledge of local people on the uses of *P. thonningii* has shown significant (P < 0.05) relationship to age groups (being higher for elderly people). The consumption practice of respondents has significant (P < 0.05) association to the Kebeles, age, and income, with higher value for Hamer, lower aged groups, and low-income families. It provides nutritious animal feed to improve browse intake, survival, and productivity of domestic animals and also improve productivity of farm through amelioration of soil fertility. The status of *P. thonningii* has been decreasing due to agricultural expansion, collection of the species for firewood, dry fencing, and due to browsing. Respondents’ attitude and interest to maintain and conserve the species has shown a significant (P< 0.05) association to gender, wealth, and level of education. Higher interest was observed in men than women, mid-high wealth class households than low income, and people with education than uneducated.

**Conclusions:**

This study attempted to provide information on the multiple uses of *P. thonningii*. Given the key roles of *P. thonningii* for the people and the environment to improve household food security, agricultural productivity, and income sources and the threats to it, the need to protect it in natural forests and woodlands and optimize its uses in agroforestry systems is high. Serious consideration of this species will ultimately allow households to reap the benefits expected to accrue from it in the arid and semi-arid areas of Ethiopia where plants of this nature are usually rare.

## Introduction

### Background of the study

*Piliostigma thonningii* (Schumach.) Milne-Redh*.* is common in open woodland and wooded grasslands of sub-humid Africa at medium to low altitudes. It is widely distributed and found throughout tropical Africa except in Somalia [1]. In Ethiopia, it occurs in deciduous woodlands and wooded grasslands in most parts of the country [2].

*Piliostigma thonningii* is one of the most important indigenous multipurpose trees used for various purposes [3]*.* It is used as source of food for humans in Benna-Tsemay and Hamer Districts of South Omo Zone, locally known as Olofoo (Benna and Hamer) [4]. Its occurrence and uses have been studied in Quara area of north Gonder [5], and Benishangul Gumuz region [6]. *P. thonningii* is known as Monkey bread or Camel’s foot in English and Kameel Spoor in Africans [7]. Reports by Tadesse et al. [8], Amente [6], Tebkew et al. [5], and Ayenew [9] showed that *P. thonningii* is also consumed by animals as a fodder. However, in other African countries, *P. thonningii* is used for various purposes other than food. Sources show that the different parts of the tree (leaves, pods, and seeds) which are nutritious are browsed by animals including elephants, livestock, and antelopes [1, 10–13]. The leaves of *P. thonningii* are eaten and chewed by the Masai people to relieve thirst [12].

The wood is suitable for poles, firewood, charcoal, carpentry, construction, to make household utensils, and farm implements [1, 2, 10]. The bark is used to make strings, ropes, and clothes [1, 2, 11]. Different phytochemicals (such as flavonoids, tannins, alkaloids, saponins, terpenoids, anthraquinones, steroids, and volatile oils) are extracted from the parts of *P. thonningii* and used to treat various diseases, such as fever, toothache, wound healing, dysentery, respiratory ailments (bronchitis, cough, and chest complaints), snake bites, hookworm, skin infections, stomach ache, and chronic ulcers [1, 12–18]. In Tanzania, the tender leaves are chewed and the juice is swallowed to treat stomach-ache, coughs, and snakebite [19]. The roots are used to treat prolonged menstruation, hemorrhage, and miscarriage in women and also for the treatment of coughs, colds, body pain, and STDs [19].

Ethnobotanical knowledge on *P. thonningii* in Ethiopia in general and South Omo Zone in particular is not adequately documented. The present study was conducted to document the different ethnobotanical uses of *P. thonningii* and the associated indigenous botanical knowledge of the local community and to identify the management practices, and threats to its sustainability in South Omo Zone. The research answers various questions about the uses of *P. thonningii*, influence of demographic factors of the local community on the indigenous and local knowledge, uses, and threats to the sustainability of *P. thonningii*.

## Materials and methods

### Descriptions of the study area

**Fig. 1:**
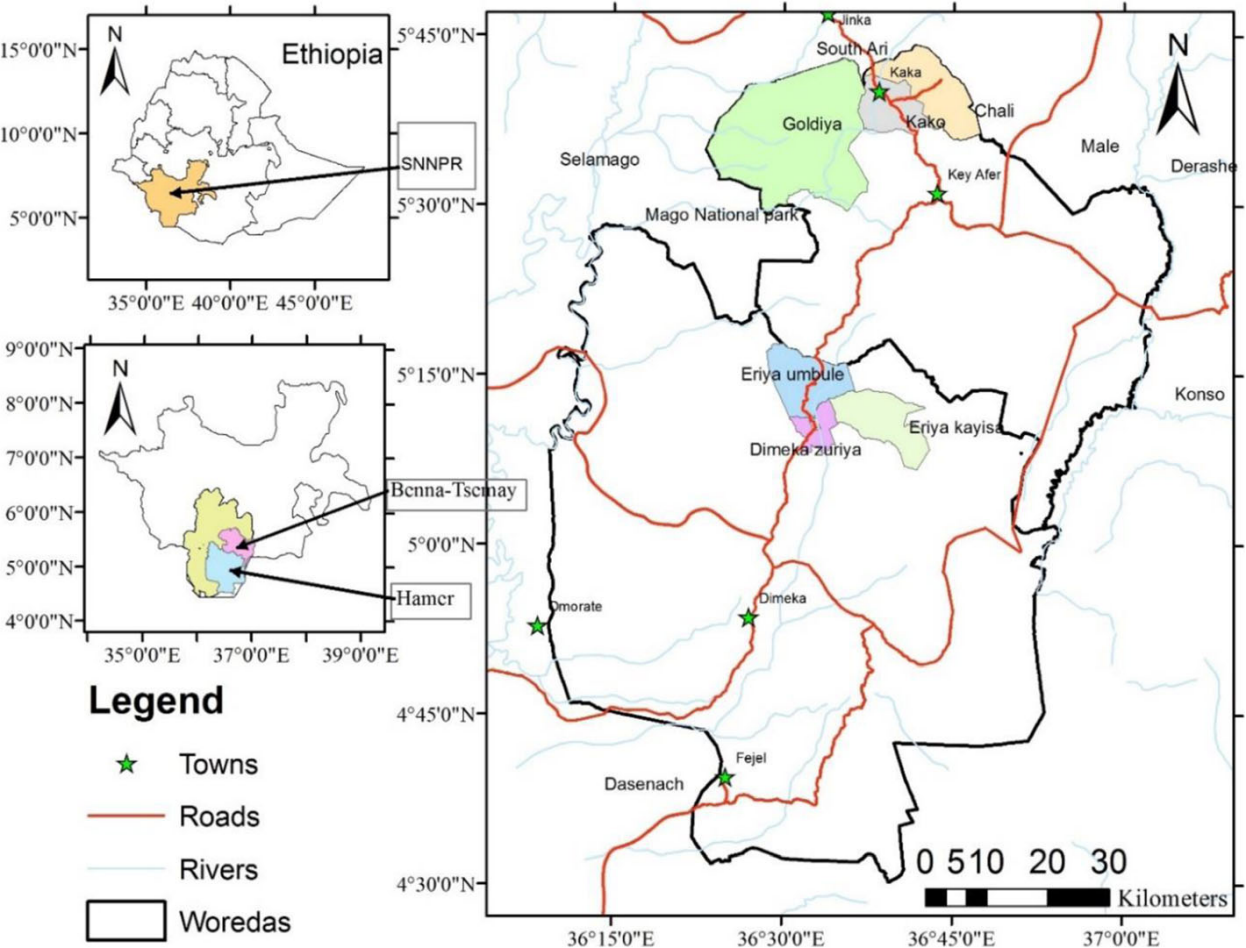
Map of Ethiopia showing the location of the study area, Hammer and Benna-Tsemay Districts in South Omo Zone, southern Ethiopia

This study was conducted in the arid and semi-arid areas of the South Omo Zone, southern, Ethiopia (Fig. 1). The South Omo zone is one of the 13 zones found in the Southern Nations, Nationalities, and People’s Regional State of Ethiopia. In the zone, there are eight districts, and the zone town is Jinka. The study districts, Benna-Tsemay (Key Afer) and Hamer (Dimeka), are located at about 739 and 839 km from the capital city of Ethiopia, Addis Ababa, respectively. Bena-Tsemay District is located at 5° 03′ to 5° 34′ N and 36° 33′ to 37° 03′ E with altitude ranging from 500 to 2000 m.a.s.l. and Hamer District is located between 4° 31′ to 5° 28′ N and 36° 09′ to 36° 53′ E with altitude ranging from 381 (Kizo-Baze plain) to 2084 m.a.s.l (Buska-Ale mountain). The study districts cover a total land area of 9496 km^2^ (Hamer = 5742 km^2^ and Benna-Tsemay = 3754 km^2^).

The greater proportion of the study area is characterized by arid and semi-arid climatic condition. According to the agroecological map of the Ethiopian Institute of Agricultural Research (EIAR [20], the agroecology of the study Kebeles (subdistricts) of Benna-Tsemay District is categorized as warm humid lowlands (H_2_) and the study Kebeles of Hamer District are categorized as warm semi-arid lowlands (SA_2_).

**Fig. 2:**
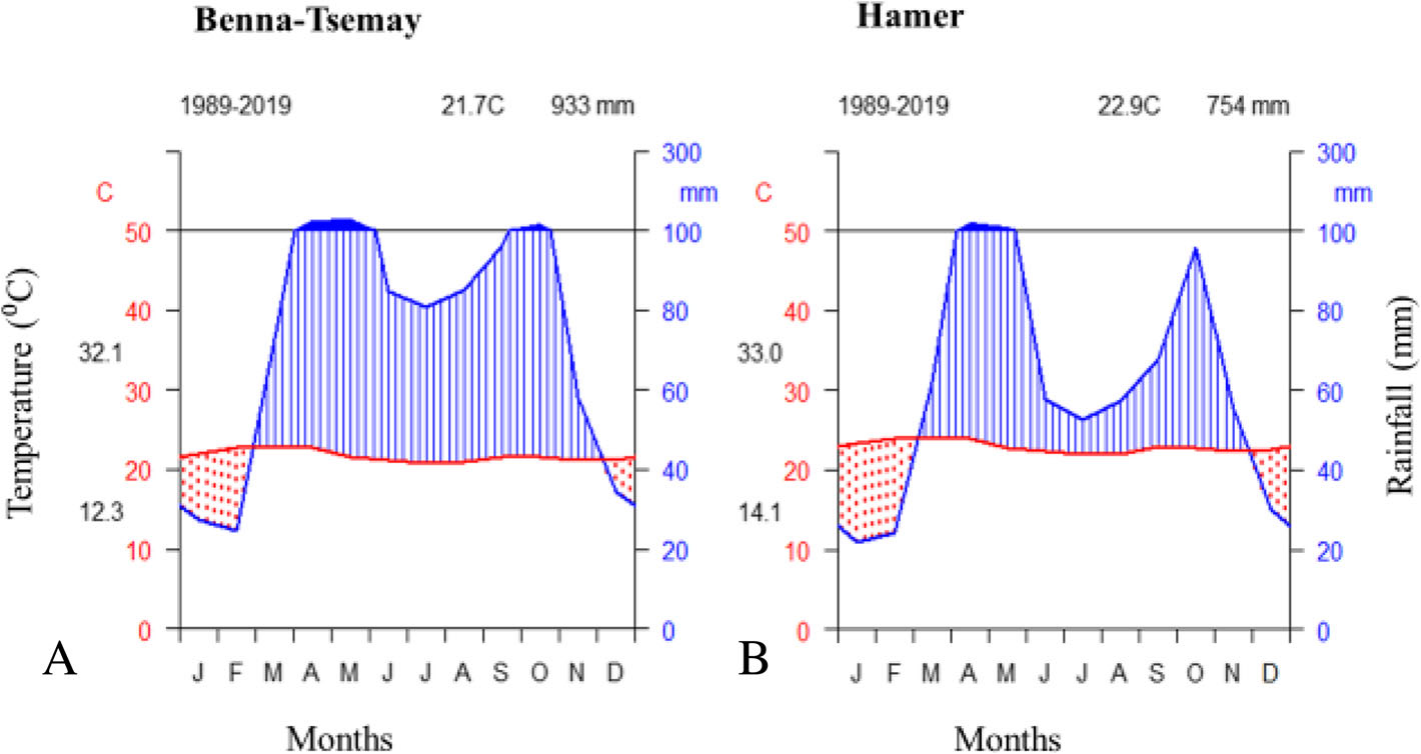
Climate diagram of the study district showing rainfall distribution and temperature variation from 1989 to 2019 [Data source: Centre for Environmental Data Analysis (Harris & Jones, 2019)]

The rainfall pattern of both districts is bimodal. The average annual precipitation of the Benna-Tsemay district was 933 mm and the average annual temperature was 20.7 °C. The dry season occurred from the beginning of December to the end of February. The long rainy period which is the cropping season occurs from the end of March to the beginning of June and the short rainy season, which is important only for pasture, occurs between October and November. The average monthly maximum temperature of the warmest month is 30.2 °C and the average monthly minimum temperature of the coldest month is 12.3 °C (Fig. 2A).

The average annual precipitation of the Hamer District was 754 mm and the average annual temperature was 22.9 °C. The dry season occurred from mid-November to the beginning of March. The long rainy season occurs from the end of March to May and the short rain season is from the end of September to the end of October. The average monthly maximum temperature of the warmest month is 33 °C and the average monthly minimum temperature of the coldest month is 14.1 °C (Fig. 2B). The climate of Hamer is relatively drier than Benna-Tsemay.

The vegetation of the study area belongs to the types known as *Acacia-Commiphora* woodland and *Combretum-Terminalia* woodland and wooded grassland [21]. Most of the pastoral and agro-pastoral communities of the study area rely mainly on dry forest resources for livestock fodder, income, food, and medicine. The majority of the communities in Benna-Tsemay District belong to Benna and Tsemay ethnic groups. The human population of the District was 55,590 [22]. However, the current (2018) report from Benna-Tsemay district agricultural bureau shows 72,749 (36,262 male and 36,487 female) (Key-Afer, Agriculture Bureau, unpublished report). The majority of the communities in Hamer District belongs to the Hamer ethnic group. The population of the District was 46,129 [22]. However, the current (2018) report from Hamer district agricultural bureau shows 78,545 (40,122 male and 38,423 female) (Dimeka, Agriculture Bureau, unpublished report). Pastoralism and agropastoralism are the two main livelihood options in the study area. The livelihoods of these pastoralist communities are mainly rearing of livestock, goats, and sheep and use of their products and the agropastoralists are dependent on both livestock products and crop cultivation. There is limited cereal crop production in Hamer and the people collect and use some wild edible plants [23]. Their main source of income was from the sale of livestock, which was followed by the sale of honey [24]. Food insecurity is a common problem in the study area caused by complex factors such as frequent and extended drought, degradation of natural resources, lack of appropriate technologies, and unpredictable climatic conditions which reduces livestock and plant productivity; this threatens the livelihoods of the pastoral and agro-pastoral communities [24, 25]. Mobility and collection of wild foods is the coping mechanisms to climate variability and food insecurity in the study area [23, 24, 26–28].

### Sampling and informant selection

A reconnaissance survey was first conducted in May 2018 to gain an overview of the availability, distribution, and consumption practice of *P. thonningii.* From the eight districts of the South Omo Zone, two districts (Benna-Tsemay and Hamer) were selected considering their difference in agroecology and presence of at least two major ethnic groups (Benna and Hamer ethnolinguistic communities) and occurrence of *P. thonningi* was taken as criteria for inclusion of kebeles in the study. A discussion was made with both Benna-Tsemay and Hamer district administrators, environment protection, forest, and climate change officers, agricultural development agents and community elders. Following this, field observation was made jointly with field assistants and agricultural development agents to select Kebeles and to check the availability, distribution, and accessibility of *P. thonningii* within the study area. The data collected through this activity helped to purposively select six sample Kebeles (three from Hamer-speaking and three from Benna speaking districts).

The sample size was determined following the standard procedure of Cochran’s sample size formula indicated in Kotrlik and Higgins [29]. Consequently, the sample sizes for the two districts were determined separately based on the total number of households in each district (11,118 Benna and 9,225 Hamer), and finally, the proportionally calculated manageable sample size for Benna-Tsemay (34 households) and for Hamer (33) was used. To make the number of the household involved in the interview equal in the two districts and in each Kebele, a total of 36 households from each district and 12 households from each Kebele were selected to make an overall total of 72 households. Informants were stratified by gender and relative socio-economic status as low wealth and medium-high wealth status households based on the information obtained from local authorities (agricultural development agents and Kebeles administrators). The wealth status of households in each Kebele was assessed by the district food security desk following the criteria set by Ministry-of-Agriculture [30], and the list of poor household who benefits from Productive Safety Net program was given to the Kebele food security task force. The 72 general informants were identified by taking one household informant from each stratified randomly identified household and two key informants from each kebele were purposively selected making a total of 84 informants. Informant sampling approach was following Martin [31] and Alexiades [32]. Thus, out of the 84 informants, 72 (12 informants × 3 Kebeles × 2 ethnic groups) were general informants selected randomly by a lottery method from the stratified households to give equal chances. The other 12 informants (from 2 ethnic groups × 3 Kebeles × 2 key informants) were key informants selected purposively based on the recommendations from local authorities and elders, confirmed to be knowledgeable about *P. thonningii* and volunteered to participate in the study.

### Data collection

The ethnobotanical data were collected using a structured interview, semi-structured interview, and direct field observation and focus group discussions [31, 33]. The structured interview was based on fixed questions and was mainly used for the collection of local knowledge. The structured questionnaire was also documented by interviewing informants using the Open Data Kit (ODK collect version 1.15.1), an application loaded on a smartphone. Each interviewee was asked the same questions independently without contacts or sharing information with the other informants or with the target population. The semi-structured interview was conducted based on a checklist of questions and both the structured and semi-structured interviews were prepared in English and translated to the local languages (Benna and Hamer) by a proficient local translator. The structured questionnaire is composed of closed questions while the semi-structured questionnaire had open-ended questions. Informants were allowed to raise their own points during the interview, not necessarily as responses to questions. The interviewer probed deeper by asking additional questions whenever interesting information or ideas were sensed.

Guided field walk procedure was followed to collect and record voucher specimens of plants found near/under *P. thonningii* in the selected Kebeles jointly with a field assistant and a translator. Field assistants were selected based on their willingness, ability to walk long distances, general plant knowledge in local name, and capability of translating both ways. The field observation created an opportunity to make a note of the relationship of *P. thonningii* with other species and other features of the study area.

Six focus group discussions (FGDs) were conducted for crosschecking and verification of the information. Accordingly, the FGDs were undertaken in groups of six in each of the selected Kebeles. The discussion was conducted with key informants, farmers, Kebele administrator, and agricultural development agents, mediated by the researcher. Information collected through focus group discussion is important to triangulate the ethnobotanical data collected through the structured and semi-structured interviews.

### Data analyses

Statistical Package for Social Sciences (SPSS) Version 26.0 was used to analyze ethnobotanical data. A Pearson’s chi-square test was used to detect (at *P* < 0.05) the relationship of the ethnobotanical knowledge held by respondents between different ethnic background, gender, wealth status, age groups, educational level, and Kebeles, regarding the use categories, consumption practice, and attitude of the respondents to conserve the species, and also used to detect the relationship between the different age groups of low- and mid-high wealth status respondents who were involved in the collection and consumption of the fruit. Ethnobotanical ranking methods were employed to analyze the most preferred use of *P. thonningii* and the most destructive threats to it*.* Direct matrix ranking was carried out to rank the most preferred use of *P. thonningii* in the study area. The 12 key informants were requested to compare the uses of *P. thonningii* based on their preference and importance to the community. Each key informant arranged the uses according to personal preference, perceived importance to the community on a 0 to 5 scale with 0—no value, and 5—the highest value. The12 key informants were requested to rank the uses of *P. thonningii*. Pair-wise ranking was employed to determine the degree of destructive activities or threats to *P. thonningii* on a scale of 0 to 5 with 0—not destructive, and 5—the most destructive. Finally, the given numbers were summed up for all key informants, giving an overall rank in the manner shown by Martin [31]. Then, the highest scores for use diversity ranking were considered as the most preferred use of *P. thonningii* to the local people and the highest scores for threats were considered the most destructive threats to *P. thonningii*.

## Results

### Demographic characteristics of Informants

A total of 84 (72 general and 12 key) informants were involved in the ethnobotanical study of *Piliostigma thonningii* and 6 individuals in each kebele participated in the focus group discussions for triangulation of the collected information. The proportions of male and female informants were equal in number (42, 50% each). All the interviewed informants were aged 18–78 years and classified into four age groups based on the perception/cognition of informants [34], demographic structure, and motivation of households [35, 36]. Fourteen percent were 18–30 years old, 35% were 31–45 years old, 45% were from 46 to 60 years old, and the remaining 6% were 61–78 years old. Respondents were from different educational backgrounds and classified following Ethiopian education system 4-4-2-2 (grades 1 to 4, grades 5 to 8, grades 9 to 10, and grades 11 to 12). Eighty percent of the respondents were illiterate, 1% able to read and write, and the remaining 8% and 11% were from grades 1 to 4 and 5 to 8, respectively (Table 1).

**Table 1:** Demographic characteristics of informants

Age groups	Gender				Educational status	Gender			
	Male	Female	Total	%		Male	Female	Total	%
**18–30**	7	5	12	**14**	Illiterate	30	37	67	**80**
**31–45**	16	13	29	**35**	Read and write	0	1	1	**1**
**46–60**	14	24	38	**45**	Grade 1 to 4	4	3	7	**8**
**61–78**	5	0	5	**6**	Grade 5 to 8	8	1	9	**11**
**Total**	42	42	84	**100**		42	42	84	**100**

### The multiple uses of *P. thonningii*

**Fig. 3:**
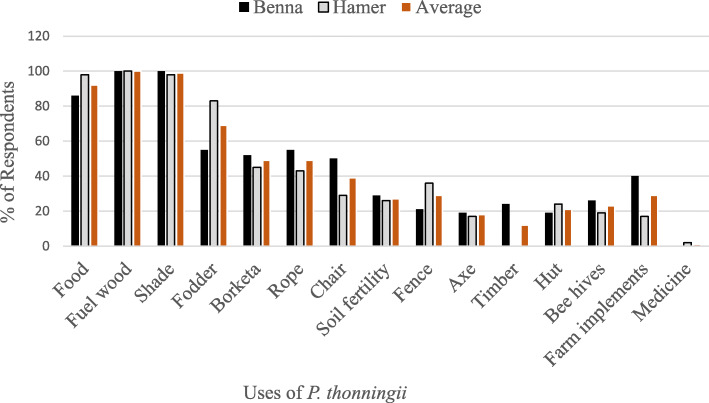
Percentage of informants’ responses on the ethnobotanical uses of *P. thonningii* (n=84)

The results of the study show that *P. thonningii* is a multipurpose tree used by the local communities for various purposes. Based on the consensus points of the focus group discussions, *P. thonningii* is recognized for its contribution to people’s livelihoods in many different ways. Both the Benna and Hamer ethnic groups use *P. thonningii* for multiple purposes*.* It provides various functions including economic and ecological functions such as provision of shade, food, fodder, soil fertility, provision of firewood, medicine, rope (cordage), *Borketa* (traditional multipurpose material used as a stool and head support), construction of huts, beehives, fences, and preparation of farm implements. Almost all respondents of both districts widely expressed that they use *P. thonningii* for firewood and shade. About 92% of the respondents confirmed that they eat the seeds and 69% verified its use as fodder for livestock, and 49% for making *Borketa* and other uses as indicated in Fig. 3.

The Hamer people predominantly use this species as a source of food (98%), fodder (83%), fence (36%), and for building huts (24%) as compared to the Benna ethnic group who reported using it predominantly for making *Borketa* (52%), rope (55%), chair (50%), timber (24%), beehives (26%), and farm implements (40%). All the uses of the species reported in the two districts are given in Fig. 3. However, the use of *P. thonningii* for fuelwood (100%), shade (100/98%), soil fertility (29/26%), and axe handle (19/17%) were comparable between the two ethnic groups. Twenty-four percent of Benna respondents indicated that they use *P. thonningii* for timber while it is not known as a timber tree in Hamer. Two percent of Hamer respondents indicated that they use it to treat ailments, while the Benna respondents did not indicate any use of it for medicinal purposes.

There is a significant relationship between the age of respondents and their ethnobotanical knowledge of *P. thonningii* (Χ^*2*^ = 273.6, df = 42, *P* < 0.0001). Older age respondents know more about the uses of *P. thonningii* than younger age respondents. There is no significant (*P <* 0.05) relationship between the ethnic groups, gender, wealth status, and educational level of the respondents regarding their knowledge on the uses of *P. thonningii*. The different use categories in each Kebele (Table 2) was analyzed using Pearson’s chi-square test and the result shows that there is a significant association (*P <*0.05) between Kebeles in the ethnobotanical knowledge of use categories (Χ^*2*^ = 107.4, df = 70, *P* < 0.003).

**Table 2:** The different use categories of *P. thonningii* in each Kebele

Kebele	Use categories														
	F	Fw	Sh	Fd	B	R	Ch	Sf	Fn	A	T	H	Bh	Fi	M
Kako	12	12	12	11	1	6	0	2	0	0	0	1	0	0	0
Goldiya	12	12	12	12	10	9	6	4	2	1	0	0	1	2	0
Cali	6	12	12	4	5	4	8	0	1	1	3	1	3	3	0
Dimeka zuriya	11	12	12	5	3	0	2	0	3	1	0	4	2	1	1
Eriya Umbule	12	12	12	12	9	6	2	3	1	0	0	0	0	0	0
Eriya Kayisa	12	12	11	12	4	5	2	2	5	0	0	0	0	0	0

*NB*: *F* food, *Fw* fuelwood, *Sh* shade, *Fd* fodder, *B* Borketa, *R* rope, *Ch* chair, *Sf* soil fertility, *Fn* fence, *A* axe, *T* timber, *H* hut, *Bh* beehives, *Fi* farm implement, *M* medicine

### The contribution of *P. thonningii* as a source of food

**Fig. 4:**
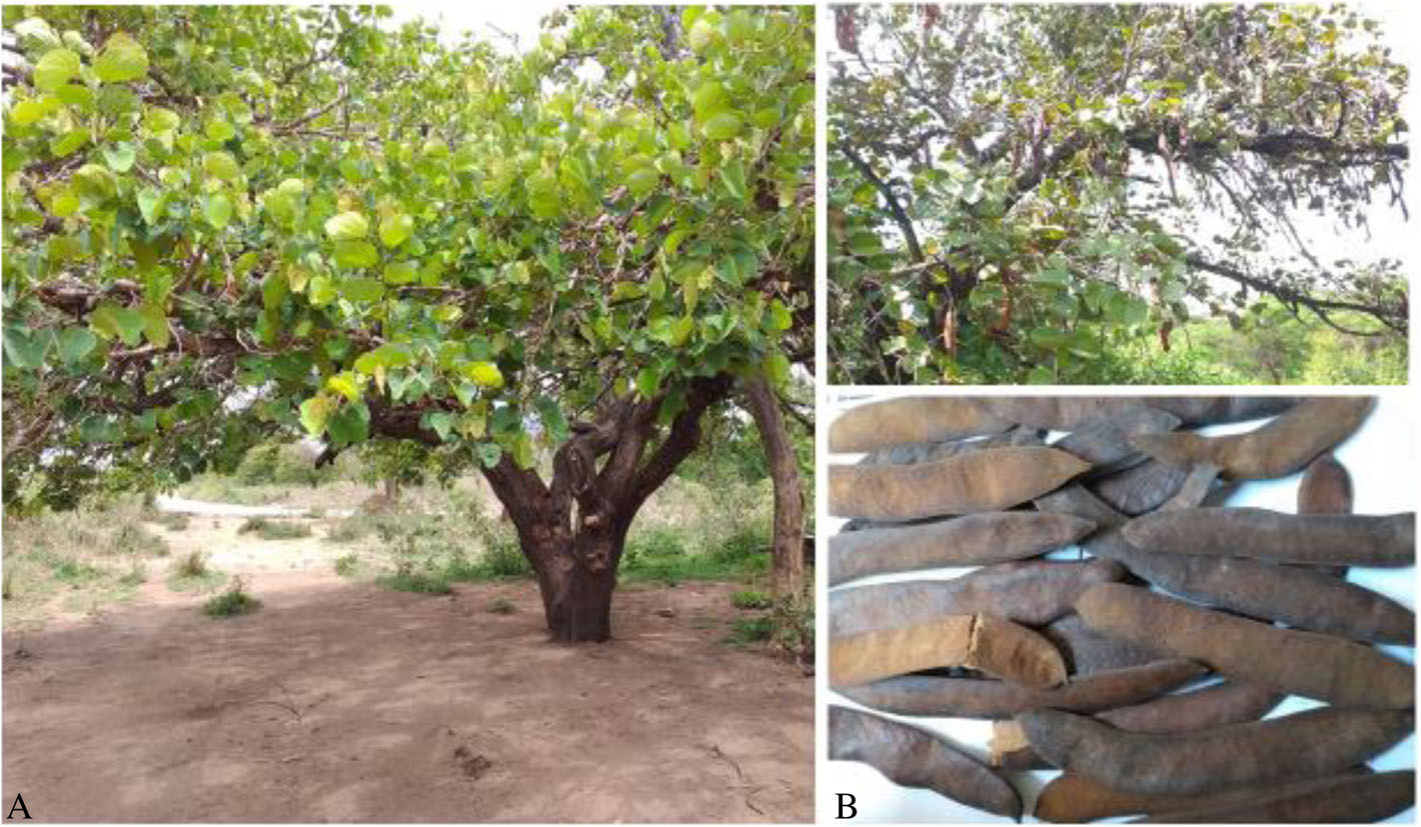
Whole tree of *P. thonningii* (**A**) and its ripened fruits (**B**)

The majority of the respondents (98% Hamer and 86% of Benna) indicated that they use *P. thonningii* as a source of food and consume the fruit during the normal time and during periods of famine to ensure food security. A study by Assefa and Abebe [4] shows that *P. thonningii* is used as a source of food and the consumption of wild food plants in the study areas ranked second as a coping mechanism for surviving during a famine period. Recurrent drought and shortage of food are serious problems of these agropastoral communities. In addition to household consumption, it provides an opportunity to supplement household incomes by selling the edible fruits when there is shortage of food [4]. The fruit ripens in December to February depending on the availability of rainfall. When the brown pod cracks and the seeds shatter away, the pulp surrounding the seeds is eaten raw mainly by children as a snack or as emergency food (Fig. 4).

### Genders and age groups that collect/consume the fruits of *P. thonningii*

The edible part (fruit) is collected from the grazing lands and farmlands and consumed raw by all community members (children, adults, and elders) during normal times and times of famine. However, 89% of the respondents reported that children were involved in the collection and consumption of the fruit while only 67% of adults and 6% of elders collect and consume *P. thonningii* fruits. There is no difference in the collection/consumption of the fruit between gender of the same age groups.

Respondents from mid-high wealth status households replied that children were involved more in the collection and consumption of the fruit than adults and elders. However, the low wealth status households replied that there is no difference in the collection and consumption of the fruit among the different age groups of poor families. The chi-square test results of the different age groups and wealth classes of informants who were involved in the collection and consumption of the fruit shows that there is a significant (Χ^2^ = 5.063, df = 1, *P* < 0.024) relationship among the respondents of low wealth status and mid-high wealth status respondents, indicating the former group consumes more. On the other hand, households in both wealth categories reported that there is no difference in the collection and consumption of the fruit between adults and elders of both low and mid-high wealth status households.

The Pearson’s chi-square test results regarding the frequency of consumption practices of respondents showed that there is a significant (*P* < 0.05) association between ethnic groups (Χ^2^ = 8.87, df = 2, *P* < 0.012), age (Χ^2^ = 13.67, df = 6, *P* < 0.034), and wealth status (Χ^2^ = 11.33, df = 2, *P* < 0.003), whereas gender and educational levels did not show significant association at *P* < 0.05. Members of the Hamer ethnic group have a strong attachment and hence consume the fruits of *P. thonningii* than the Benna ethnic group and poor households consume more than the rich households. However, there is no significant difference between the Kebeles in the frequency of the consumption of the fruit. The consumption of the fruits does not have a complex negative side effect, though 17% of the respondents reported that they experienced side effects, such as stomach aches, loss of appetite, and constipation, while 83% of the respondents did not complain of any side effect as a result of consumption of *P. thonningii* fruits.

### The role of *P. thonningii* as a source of livestock feed

The use of *P. thonningii* as livestock feed is common in both (Benna-Tsemay and Hamer) communities. The majority of the respondents (83%) reported that they use *P. thonningii* as a source of feed for their livestock. However, some (17%) of the respondents replied that they do not use it as livestock feed. All of the respondents of both study areas reported that *P. thonningii* is used as a preferred livestock feed both during times of feed availability and during the dry season. The browsed parts of *P. thonningii* are mainly seeds, fruits (pod), and leaves, especially the shoot and young leaves are copiously consumed by animals. The majority of the respondents (83%) indicated that seeds and fruits (pods) are the most consumed parts than any other parts. However, 69% of the respondents replied that leaves are also consumed by livestock (cattle, goats, and sheep). Small branches are also lopped as dry season fodder for goats and the branches are used to reinforce fences.

Respondents of both districts reported that different livestock types (cattle, goats, and sheep) consume the parts of *P. thonningii.* The consumption of *P. thonningii* by cattle, goats, and sheep depends on the availability of feeds. The majority of respondents reported that it is preferred and consumed by cattle (83%) and goats (80%), and few respondents replied that it is consumed by sheep (15%).

### Agricultural values of *P. thonningii*

Respondents revealed that *P. thonningii* is used as an agroforestry tree to improve agricultural productivity. Almost all of the respondents (100% of Benna and 97% of Hamer) reported that they use the species as a shade tree for their crops and animals and 17% of Benna and 14% of Hamer respondents reported that soil under and near *P. thonningii* is moist and fertile. They also use the tree for making farm implements; 40% of Benna and 17% of Hamer ethnic groups reported that they use the species to make different farm materials. Respondents explained why they prefer to cultivate crops under *P. thonningii* where 88% of the respondents reported that they cultivate crops near/under *P. thonningii* by saying that it controls the temperature and keeps the soil moist, 74% of the respondents reported that it provides good shade for their crops and animals, and the other 29% of the respondents reported that it makes the soil fertile.

During the field survey, the researcher observed and recorded different herbaceous and woody plant species growing under/near (up to 1 m radius outside the tree canopy) *P. thonningii* trees that added to 29 species distributed in 18 families. This finding indicates that *P. thonningii* creates a conducive environment for establishment and perpetuation of different plant species belonging to different families under its canopy or near it. The woody species constituted 69% (45% shrub, 24% tree species), herbs constituted about 20%, and climbers constituted 10%. Further analysis of the data showed that 7 (25%) species belong to the family Fabaceae, two species each to Anacardiaceae, Euphorbiaceae, Rubiaceae, Rutaceae, and Tiliaceae and a single species each in the remaining 12 families (Appendix 1). Many of the characteristics of *P. thonningii* showed that it is an ideal agroforestry tree.

**Fig. 5:**
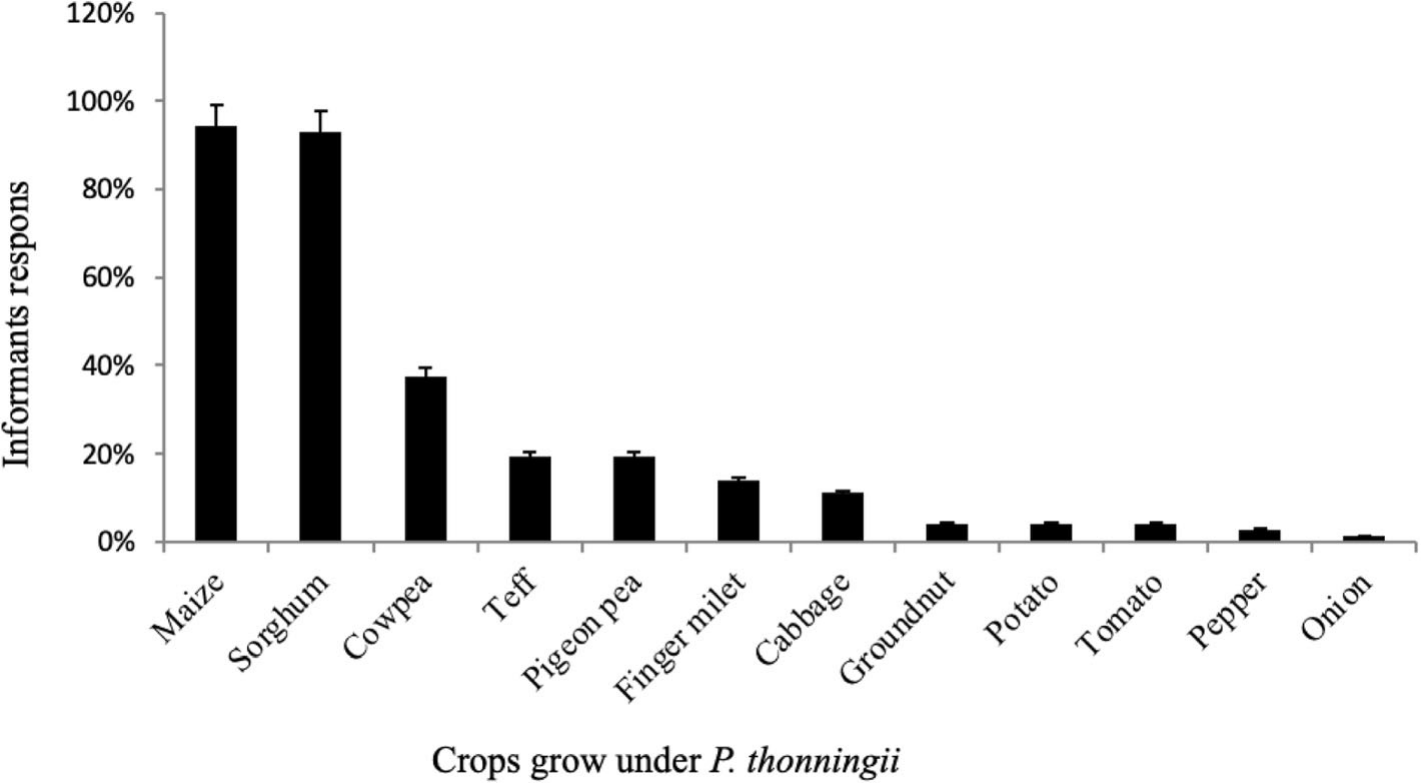
Percentage of farmers’ response on the type of crops grown under/near *P. thonningii* in Benna-Tsemay and Hamer districts (n=84)

The results of the study further showed that members of the local community cultivated different crops near/under *P. thonnongii* including maize (*Zea mays* L*.*), sorghum (*Sorghum bicolor* L.), cowpea (*Vigna unguiculata* (L.)), Teff (*Eragrostis tef* (Zucc.)), pigeon pea (*Cajanus cajan* (L.)), finger millet (*Eluesine coracana* (L.)), cabbage (*Brassica* spp.), groundnut (*Arachys hypogea* L.), potato (*Solanum tuberosum* L.), tomato (*Lycopersicon esculenta* L.), pepper (*Capsicum annuum* L.), and onion (*Allium cepa* L.). The responses of the majority of the respondents showed that the crops most abundantly and commonly cultivated under/near *P. thonningii* are maize (95%), sorghum (94%), and cowpea (45%) (Fig. 5)*.*

### The role of *P. thonningii* as a source of fuel and other uses

The results of the study show that *P. thonningii* is used as a source of fuel, household materials, and other uses. The woody part of the plant is used as a source of firewood, for making *Borketa*, hut, beehives, chair, fence, axe handles, and timber. Regarding firewood collection, all (100%) of the respondents from both districts agreed that they use the wood as a source of fuel, 49% of the respondents reported that wood is used for making household materials such as *Borketa*, a traditional multipurpose material used as a stool and head support (the structure of *Borketa* is found in dryad data repository); 39% for chair; 29% for fencing; 23% for making beehives; 21% for making huts; 29% for farm materials; 18% for axe; and 12% for timber. About 49% of the respondents reported that the inner layer of the bark is used to make rope and 1% of the respondents reported that the bark is used as traditional medicine for the treatment of liver disease through fumigation.

The results of direct matrix ranking, which was carried out to evaluate the multipurpose uses of *P. thonningii* and their relative importance to the local people, showed that fodder, food, and firewood ranked in the 1st, 2nd, and 3rd places respectively. And the remaining uses of *P. thonningii* as a shade tree, agricultural value, fencing, making household and farm materials, beehives, and medicine take the ranks from 4 to 9 respectively (Table 3). This shows that the local people use *P. thonningii* mainly for fodder, food, and fuelwood; however, they also use it for different purposes, indicating that it is a multipurpose tree.

**Table 3:** Direct matrix ranking of the use preferences of *P. thonningii* by 12 key informants

Uses	Key informants													
	K1	K2	C1	C2	G1	G2	D1	D2	U1	U2	Ky1	Ky2	Total	Rank
Food	4	3	3	3	4	4	3	3	4	3	4	4	42	2
Shade	3	3	2	2	3	3	2	3	3	3	3	2	35	4
Fodder	4	4	4	4	5	4	4	3	5	4	5	4	50	1
Firewood	3	3	2	4	3	4	3	3	3	4	3	4	39	3
Agriculture	2	2	3	3	2	3	3	1	2	2	2	0	25	5
Beehives	1	0	2	0	2	1	0	0	2	0	2	0	10	8
Fencing	3	2	0	2	1	2	1	2	2	3	3	2	23	6
Medicine	0	0	3	0	0	0	0	0	0	2	0	0	5	9
Household & Farm materials	1	2	2	1	0	1	2	1	2	1	2	2	17	7
**Total score**	21	19	21	19	20	22	18	16	23	22	24	18	246	
**Kebele average**	20		20		21		17		22.5		21			

*NB*: *K* Kako, *C* Cali, *G* Goldiya, *D* Dimeka zuriya, *U* Eriya Umbule, *Ky* Eriya Kayisa

### The status of *P. thonningii* in the study area

**Fig. 6:**
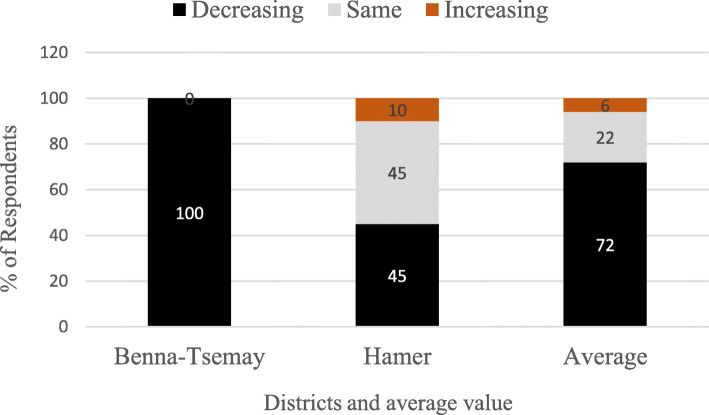
Informant’s response on the status of *P. thonningii* in the study area (72)

All respondents from Benna and 45% of the respondents from the Hamer District reported that the abundance of *P. thonningii* has decreased while the other 45% of respondents from the Hamer District replied there is no difference in abundance as observed over time and while a minority (10%) of the respondents from Hamer District felt that the abundance of *P. thonningii* has increased in the past 10 years (Fig. 6).

### Threats to the sustainability of *P. thonningii*

All respondents of Benna and the slight majority (55%) in Hamer reported that the sustainability of *P. thonningii is* threatened by some unfavorable factors. However, 45% of Hamer respondents replied that there are no threats to the sustainability of *P. thonningii*. According to the information from respondents and participants of the focus group discussion, the main threatening factors to *P. thonningii* are agricultural expansion, firewood collection, overgrazing, harvesting for construction of fences, use for farm and household materials, and charcoal production. Based on this information, the degree of destructive effects of these threats was analyzed using pair-wise ranking. The results suggested that agricultural expansion, firewood collection, fencing, overgrazing, making household and farm materials, and charcoal production are the major factors threatening the continuity of *P. thonningii* in the two districts. Agricultural expansion and firewood collection ranked in the 1st and 2nd places, respectively, indicating that they are the most proximate threatening factors (Table 4).

**Table 4:** Results of a pair-wise ranking of factors considered threats to *P. thonningii* and their degrees of destructive effects

Threatening factors	Respondents of each Kebele													
	K1	K2	C1	C2	G1	G2	D1	D2	U1	U2	Ky1	Ky2	Total	Rank
Firewood	4	4	4	4	5	4	4	4	5	5	4	5	52	2
Agricultural expansion	5	5	5	5	4	5	4	5	4	4	5	4	55	1
Overgrazing	2	2	3	1	3	2	1	2	2	2	2	3	25	4
Fencing	3	2	2	3	2	3	2	3	3	1	3	2	29	3
Household and Farm materials	1	1	1	2	2	1	0	1	1	2	2	1	15	5
Charcoal	0	0	1	0	1	1	1	0	0	0	0	0	4	6

*NB: K* Kako, *C* Cali, *G* Goldiya, *D* Dimeka zuriya, *U* Eriya Umbule, *Ky* Eriya Kayisa

### Traditional management practices and the attitudes of respondents to maintain and conserve *P. thonningii*

**Fig. 7:**
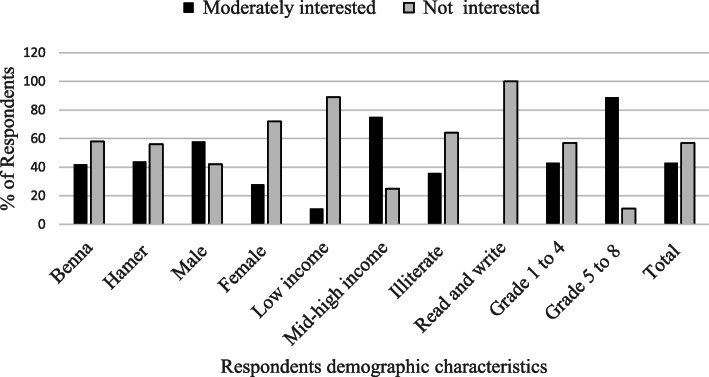
The attitude of respondent’s to maintain and conserve *P. thonningii* (72)

Respondents’ perceptions about the management and conservation practices of *P. thonningii* showed that 89% of the low wealth status and 25% of mid-high wealth status responded that they do not have awareness and interest to manage and conserve *P. thonningii*. However, 11% of the low wealth status and 75% of mid-high wealth status respondents expressed that they are interested and willing to maintain and conserve the species. Over half of the male respondents (58%) and 28% of female respondents replied that they are interested to maintain a good cover of *P. thonningii* but 72% of female and 42% of male respondents were not interested and lack the willingness to manage and conserve. The educational level also affects the perception and willingness to manage and conserve. Most of the respondents had formal education, grades 5 to 8 (89%), and 36% of the illiterate respondents expressed their interest to maintain and conserve *P. thonningii* but 64% of the illiterates, all of those who can only read and write, and 11% of those who had formal education level of grades 5 to 8 do not show an interest to maintain and conserve *P. thonningii* (Fig. 7).

The Pearson’s chi-square test results on the perception and attitude of respondents to maintain and conserve *P. thonningii* shows that there is a significant association between gender (Χ^2^ = 18.36, df = 1, *P* < 0.0001), wealth status (Χ^2^ = 83.56, df = 1, *P* < 0.0001), and educational level (Χ^2^ = 71.17, df = 3, *P* < 0.0002) of respondents. However, there is no statistically significant (*P* < 0.05) association between the different age and ethnic groups regarding their perception and attitude towards the maintaining and conserving of *P. thonningii*. This indicates that men, mid-high wealth status households, and educated people are more knowledgeable and have willingness to maintain and conserve *P. thonningii* than women, low wealth status households, and uneducated community members. The statistical analysis result shows that there is no significant difference between kebeles towards the maintaining and conservation of *P. thonningii*. They manage and conserve the species in their farms and homesteads by avoiding cutting and protecting from livestock damages.

## Discussion

### The multiple uses of *P. thonningii*

The results indicate a strong relationship between the local communities and *P. thonningii* in that the species provides several economic and ecosystem functions in the study area so much that it has become an integral part of the local culture. This shows that there is an intrinsic relationship between humans and their environment and between knowledge about plants and their uses. In this regard, Martin [31], Kacholi [37], and Amjad and Arshad [38] describe the relationship between plants, people, and the environment and the knowledge of local communities about the uses of plants as a source food, medicine, fuelwood, timber, fodder, shade, poles, and habitats for other organisms. Similarly, Demel et al. [39] describe the indirect benefits of wild edible plants such as sources of genetic diversity; encourages agroforestry practice in dry land areas; habitat for different organisms; rehabilitation of degraded lands; and soil and water conservation as well as mitigation and adaptation to climate change. The ethnobotanical knowledge of respondents about *P. thonningii* among the two ethnic groups within and between the different wealth status, gender, and educational level is similar. This indicated that the transfer of indigenous knowledge on the uses of *P. thonningii* within the community is not differentiated by wealth status, gender, and level of education because of the co-existence and similarity of their cultural practices and the pastoralist mode of life. This finding is in line with that of Awas et al. [40] that showed the ethnobotanical knowledge of Berta and Gumuz people of western Ethiopia equated with the age and educational level. The relationship in the knowledge of respondents on the uses of *P. thonningii* among the different age groups showed that older members of the communities were more knowledgeable on the uses of *P. thonningii* as compared to the younger member of the communities. The relationship in the ethnobotanical use of *P. thonningii* between Kebeles associated with the life style of the households, food insecurity, availability of infrastructure, and distance of the Kebeles from the town. For example in relation to food and timber, Cali Kebele was different from others because majority of the people of Cali involved in crop cultivation other than using wild plants. In the use as a fodder, Cali and Dimeka zuriya Kebeles were different from other Kebeles because these Kebeles are relatively near the town and involved in agriculture crop production and use crop residue for their livestock feed. In agreement with this, Bortolotto et al. [41] describe that proximity to urban centers reduces the use of wild plants for various purposes. The Kebeles Goldiya and Eriya Umbule use the tree for making *Borketa* more than the other Kebeles, because majority of the communities were pastoralist, they move from place to place to find animal feed during drought and use the material as a stool and head support.

### Contribution of *P. thonningii* as a source of food

A study by Teklehaymanot [42] shows that people in semi-arid and arid areas rely more on wild edible plants as compared to people in humid and highland regions. This study was conducted in the arid and semi-arid areas of southern Ethiopia where there are harsh environmental conditions. In arid and semi-arid environments drought has a dominant effect on agricultural food production and livestock productivity. In most arid environment, the role of wild edible plants as food security agents has been reported by different authors [4, 26, 43–45]. The results of the present study showed that the communities of the South Omo Zone consume *P. thonnngii* as a supplement to staple foods and to fill a variety of food gaps besides fighting food insecurity during period of drought. Similar studies by Guinand and Lemessa [26], Terefe et al*.* [24]; Addis et al*.* [23], and Kidane and Kejela [27] indicated that the collection of wild foods is used as coping mechanisms to climate variability and food insecurity. A higher use of wild foods has been linked with greater food security [46]. Thus wild edible plants served as “buffer food” as they had rescued people during floods, drought, and major famines. In agreement with this, the finding of Hidosa et al*.* [47] and Teklehaymanot [42] indicated that people in semi-arid and arid regions of the Oromia and Afar region survive times of severe hunger by consuming wild edible plants. The fact that the fruit is the edible part of *P. thonningii* and consumed raw has an added advantage as an emergency quick fix facility at critical moments in the daily and annual cycles. This also goes along with the general finding in Ethiopia that the fruit is the dominant parts of wild edible plants [23, 28, 45, 48, 49]. *P. thonningii* is relevant to household food security to supplement the staple food in normal times, to fill seasonal food shortages, and for fighting poor micronutrient intake or otherwise called hidden hanger [50]. Similar studies in Africa show that the fruit and seeds of *P. thonningii* are nutritious and contain the primary metabolites like protein, starch, fat, and lignin [11–13, 15]. As reported by Poulton and Poole [51], Awas [52], and Adhikari et al*.* [53], wild edible plants are nutritionally rich and supply vitamins and micronutrients. In addition to improving food security, the consumption of fruits from trees improves blood circulation, prevents diabetes, and reduces obesity, cancer, and chances of being affected by heart diseases [54]. The finding on raw consumption of the fruits of *P. thonningii* equates to the work of Guinand and Lemessa [26], Balemie and Kebebew [49], Belayneh et al*.* [55], Teklehaymanot [42], and Dejene et al. [56], indicating fruits of most wild edible trees are consumed raw, not cooked or preserved, suggesting that the fruit is not toxic.

The difference in ethnic background, age, and wealth status of the community has affected the pattern of collection and consumption of the fruit. The mid-high wealth status families tend to see the consumption of the fruit of *P. thonningii* as a sign of poverty, but poor families collect and consume the fruit to mitigate the risks of hunger and make an important part of their daily dietary intake. In agreement with this, Poulton and Poole [51] reported that wild edible plants make many contributions to poor people’s livelihoods and most of the disadvantaged and vulnerable peoples of the society mitigate risks using wild food plants. However, in the study area when there is a serious shortage of food affecting both the poor and rich families, all are involved in the collection and consumption of the fruit as explained by informants.

According to mid-high wealth status respondents, children are the main participants in the collection and consumption of the fruit than adults and elders. This is because children can climb up to a tree, collect the fruit while tending cattle in the grazing fields, fetch water, collect firewood, or walk to the fields. On the other hand, adult members of the community mostly spent their time on other daily activities like farming, the market, and other social activities. Moreover elder members of the communities spend most of their time at home and do not have a chance to get and collect the fruit. However, during food shortage adults and even elder members of the community are involved in the collection and consumption of the fruit. In agreement with this, the findings of Guinand and Lemessa [26] and Teklehaymanot and Giday [45] indicated that adults and elders collect and consume the fruit from the wild and over 70% of the wild edible plants were consumed during times of food scarcity and starvation. In normal times, mainly children and poor families were involved in the collection and consumption of the fruit. This goes in line with the response of low-wealth status households who stated that there is no difference in the collection and consumption of the fruit among the different age groups of poor families.

The consumption of the fruit is sometimes accompanied with some complaints. A few respondents (17%) reported that excessive consumption of the fruit causes stomach aches, loss of appetite, and constipation and induces thirst. Other than minor complaints and discomforts, *P. thonningii* seeds or other parts of the plant did not cause serious side effects to the majority of the respondents (83%) even when consumed uncooked. This may be related to the assertion by Nwosu et al. [57] that the level of dietary fiber of *P. thonningii* is quite high when compared with that of most legumes and seeds. Dietary fiber plays an important role in reducing the risk and lowering the incidence of numerous diseases [58]. It prevents constipation and provides other health benefits, such as maintain a healthy weight and lowering the risk of diabetes, heart disease, and some types of cancer. This indicates fiber-rich food is suitable for health and the local people know the importance and contribution of wild fruits to their daily dietary ratio as well as alleviating possible health problems such as stomach irritation, stomach aches, and constipation.

### The role of *P. thonningii* as a source of animal feed

The majority of the respondents (81% of Hamer and 75% of Benna-Tsemay Districts) replied that the seeds, fruit (pod), and leaves of *P. thonningii are* consumed by different livestock types. It is an important fodder tree in the study area with the leaves, pods, and seeds being nutritious and preferred by cattle, goats, and sheep. It is browsed directly from the tree or lopped/cut fresh during feeding. The edible parts provide valuable livestock feed under open grazing conditions. This is especially important during the dry season when grazing resource is limited. The use of the parts of *P. thonningii* as livestock feed has been reported in different studies such as Guinand and Lemessa [26], Geta et al*.* [59], and Ayenew [9]. In agreement with this study, the findings of Jimoh and Oladiji [13], Bekele and Tengnäs [3], Orwa et al. [1], Egharevba and Kunle [11], Chidumayo [10], and Jemiseye et al. [12] revealed that the pods are nutritious and preferred by domestic animals such as cattle and browser wild animal species such as the African elephant and antelopes.

The *P. thonningii* leaf meal has high crude protein content (20.5%) [57], as compared to other browse trees (like finger millet straw) indicating the potential of this tree leaf to be used as a supplement to improve poor quality feed. In the study area, livestock prefer the young leaf over the matured one in agreement with Nwosu et al. [57] that reported higher crude protein content of *P. thonningii* at the early stage of the leaf than the mature leaf. A similar study on the use of multipurpose fodder trees by Geta et al*.* [59] indicated that *P. thonningii* leaves are nutritious and improve poor-quality roughage, and are important for livestock production in promoting health, growth, and milk production. The proper use and management of *P. thonningii* is, therefore, essential to realize these potentials.

### Agricultural uses of *P. thonningii*

Integrating *P. thonningii* into the agricultural field and grazing land plays an important role in achieving household food security and in the conservation of other plants. *P. thonningii* allows good production of crops and creates a suitable microclimate for other plants growing under or near the canopy. The findings of Chidumayo [10] indicated that it is a promising agroforestry tree in sub-Saharan Africa. It is a deciduous tree that undergoes a physiological dormancy and sheds its nitrogen-rich leaves during the dry season, to cope with moisture deficiency. The litter from the shed leaves improves the soil structure and permeability while the retained leaves in the dry season provide shade to conserve the available soil moisture by reducing the evapotranspiration under the tree. The findings of Wood and Burley [60] and Chidumayo [10], which are in agreement with this study, show the role of *P. thonningii* as an excellent shade tree which helps in retaining soil moisture and nutrients. Thus, plants under *P. thonningii* remain green for a longer period in the dry season providing grazing material for both wild and domestic animals. A study by Woldu et al. [61] shows the role of canopy trees like *Acacia tortilis* and *Acacia Senegal* in promoting better growth and productivity of agricultural crops and herbaceous species growing under their canopy by creating favorable conditions to the soil nutrient and moisture.

### The role of *P. thonningii* as a source of fuel and other uses

The use of *P. thonningii* as a source of fuelwood, for construction (making huts), for making of household equipment (*Borketa*, chair, and axe handles), fences, beehives, and timber are widely experienced. In agreement with this, Thulin et al. [2], Bekele and Tengnäs [3], Orwa et al*.* [1], and Chidumayo [10] reported the suitability of *P. thonningii* as a source of fuelwood, poles, charcoal, carpentry, construction, to make household mat and farm implements. The utilization of this tree is linked with the daily life activities of the local communities of the study area. In the study area, firewood is the most preferred and affordable domestic fuel for cooking and heating for both rural and urban communities. In agreement with this study, the findings of Gebreegziabher [62] and Eastaugh et al. [63], showed the importance of multi-purpose woody species to increase the supply of fuelwood for rural household consumption and 80% of rural households use fuelwood as their primary energy source. Similarly in southwestern Ethiopia, fuelwood is the main energy source for the majority of the people [64]. And also, Bekele [65], Gebreegziabher [62], and Mekonnen and Köhlin [66] reported that more than 90% of households in Ethiopia use biomass fuels (fuelwood, animal dung, and agricultural residues) especially fuelwood as their primary energy source to meet their cooking and heating needs and more (99.9%) rural households rely on biomass fuels than those in urban areas.

The results of the study showed that 49% of the respondents reported that the inner part of the stem bark is suitable for making ropes. In the study area, ropes are used for tying the roof of the huts and to make whips for herding goats and cattle. Similarly, Thulin et al. [2], Orwa et al*.* [1], and Egharevba and Kunle [11] confirmed the use of the inner bark to make ropes/cordage. Unsustainable use for making ropes and some other functions become destructive to the species. Therefore, it is necessary to use sustainably without killing the parent tree.

Few respondents (1%) reported the use of *P. thonningii* for traditional medicinal uses for the treatment of liver disease through fumigation; similarly, a study by Kidane et al. [67] shows that the leaf of *P. thonningii* is used for the treatment of similar diseases. Inversely, a study by Awhin et al. [68] shows that chronic/habitual/recurring consumption of *P. thonningii* may cause liver injury thereby increasing the liver enzyme activity. There is no information regarding its medicinal use in the other parts of Ethiopia; therefore, there is a need for more research on the medicinal uses of *P. thonningii*. The plant is not extensively used for medicinal purposes presumably because there are other medicinal plants more potent than *P. thonningii* preferred by the people of the study area. However, in different African countries (such as Nigeria, Kenya), it is used as a traditional medicine. The different parts (flowers, bark, fruits, roots, leaves, and stems) are used elsewhere to treat various diseases [1, 11–16, 18, 57, 69]. It is important to document and share the experience to other people elsewhere about the medicinal uses of *P. thonningii* in case they are not aware of it*.*

### The status of *P. thonningii* in the study area

The abundance of *P. thonningii* is decreasing (72.6%) mainly due to expansion of agricultural land, firewood collection, overgrazing, and harvesting wood for construction of fences and charcoal production, while the others (22.6%) reported that there is no change in the status, because before 10 years the local community used to set bushfire to enhance the re-sprouting of pasture grasses and to control animal exo-parasites (tsetse fly and ticks). Currently, setting bushfire intentionally or inadvertently is prohibited which promotes the reestablishment of woody species while the expansion of agricultural land decimates the tree cover. The effects of the two opposite processes cancel each other and the status of *P. thoningii* appears to remain unchanged. However, 4.76% of the respondents indicated that the status is increasing due to the prohibition of fire setting. Reilly et al. [70] describes that, after fire setting, there is an increase in species richness of all plants, and Burkle et al. [71] reported that local species richness of forbs was higher in burned landscapes compared to unburned landscapes at the low-productivity site, but lower in burned landscapes at the high-productivity site. The researcher field observation from 2018 to 2020 confirmed that there is a decrease in the status of *P. thonningii* because of the destruction of the habitats. Many of the woodland and grazing lands were converted to agricultural land; in some areas, there is still illegal bushfire to acquire more agricultural land and the awareness of the local community to the conservation of natural resources is limited.

### Threatening factors to *P. thonningii*

*P. thonningii* faces different threats to its continued existence from various human activities. Agricultural expansion, firewood collection, and harvesting for fencing were found to be the most threatening factors. Agricultural expansion is a major threat because nowadays the community of the study area is changing from a pastoral to an agropastoral way of life and that the population is increasing. The suitable habitats for the growth of *P. thonningii* are increasingly threatened by the continued conversion of the woodland and grazing land into farmlands. A study by Assefa and Abebe [4] showed that the community of the South Omo Zone is changing from a pastoral to an agropastoral way of life. In agreement with this study, FAO [72] showed the major cause of deforestation in Ethiopia resulted from the conversion of forested land into agricultural land and other land-use systems, and Dandena [73] explains the effect of agricultural expansion on resource availability in rural areas, thereby decreasing the volume of fruits harvestable for private consumption. During the field observation and group discussion, it was observed that the agricultural development workers and local administrators push the pastoral community to involve in agriculture without selecting appropriate land suitable for agriculture and without consideration of the agroforestry system. And also, both the rural and urban households use firewood predominantly as a domestic fuel including *P. thonningii*. Similar findings were reported by Gebreegziabher [62] who indicated that multi-purpose woody species are important to increase the supply of fuelwood for rural household families. Hence, strategies should be designed to protect and domesticate these plants for future use. There is also harvesting of the tree for various uses such as for fences without protection and hence causing damage.

### Traditional management practices and attitude of respondents to maintain and conserve *P. thonningii*

The attitude and interest of respondents to maintain and conserve *P. thonningii* is different among gender, wealth status, and educational levels. Respondents from mid-high wealth status, men, and educated members of the community practice some activities to manage and conserve *P. thonningii* that grow in their farms and homesteads by avoiding cutting and protecting it from livestock damage but there is no direct planting and protection of *P. thonningii* found in their communal land (grazing and woodland). Personal observation also confirmed that *P. thonningii* grow in farmlands, farm boundaries, watershed areas, and homesteads as live fence and shade. This tradition is a good implication of indigenous management strategies for conservation and management of this species. A study by Cao et al. [74] and Kebebew and Leta [75] describes the importance of introducing the preferred wild plants into farm lands and home gardens for the conservation of biodiversity and mitigation of climate change. However, respondents from low wealth status have limited or are not interested to maintain and conserve the species, because most of the low wealth status respondents do not have their own land or have a parcel of their own land. They are using borrowed land, and in such borrowed land, they are not allowed by the owner of the land to plant perennial plants. Therefore, low-wealth status households do not have planting experiences at all, but not only that they lack the willingness to manage and conserve. Similar to this, Ayenew [9] indicates the very low farmers practice to plant and manage the tree. They are constrained by the priority to ensure basic food self-sufficiency in their allocated land and give prior satisfaction to their needs to produce staple food. A similar finding by Poulton and Poole [51] shows poor households lack lands for the cultivation of wild edible fruits and they focus on the cultivation of crops that help them to ensure their basic food requirement on their lands. The other difficulties are the low market values of wild fruits when compared to cultivated fruits, which discourage the growth of fruit by the poor households. This information shows that, as the wealth status increases the attitude of respondents to manage and conserve also increases. Similarly, as the education level increases, the knowledge and attitude to manage and conserve also increases, indicating that educated communities have more knowledge and willingness to maintain, conserve, and plant the tree*.* On the other side, men have more interest to maintain, conserve, and plant *P. thonningii* than women. In this regard, in the study area, men control and manage the land and participate in tree planting than women; this restricts female participation in planting. As indicated by Gandile et al. [76], the most notable biodiversity conservation practice was the protection of forests using indigenous knowledge. Therefore, it is necessary to incorporate economic and cultural importance in biodiversity assessments and conservation measures, involving local resource users and all stakeholders from different sectors to maintain and conserve the species. The ecological knowledge held by local and indigenous users must be recognized and fully incorporated into management and conservation plan. In agreement with this, Carvalho and Frazão-Moreira [77] describe the importance of local knowledge in plant resources management and conservation. Creating awareness on the importance of wild plants for millions livelihoods among policy makers and across the society involves the community leaders to give proactive, protective orders to community to maintain and protect useful trees.

## Conclusion and recommendations

This study attempted to provide information on the multiple uses of *P. thonningii* besides documenting the traditional knowledge of the local community. In the study area, the pastoral and agropastoral communities use *P. thonningii* for various purposes. There is a good transfer of local traditional knowledge between the communities on the uses of *P. thonningii*. However, it was observed that there is knowledge gap through generations indicating that the youth are less knowledgeable about the uses of *P. thonningii*. Therefore, documentation of the traditional knowledge of the local communities on the uses of *P. thonningii* in the present study area and other parts of the country is very valuable and needs to be scaled-up.

*P. thonningii* contributes to the nutritional intake of the local communities and helps to ensure food security during the times of seasonal food shortage. Therefore, consideration should be given to *P. thonningii* when strategies are developed to fight food insecurity and to improve rural livelihood systems. It is an excellent fodder tree, used by the pastoral and agro-pastoral communities of the study area to feed their livestock under open grazing conditions. It improves the nutritional content of poor feed quality and the survival and productivity of livestock. It is also used as an excellent agroforestry tree to improve soil fertility, moisture retention capacity, and maintain soil structure in addition to provision of human food, livestock feed and other related uses. The local communities also use it for different purposes, such as source of fuelwood, for construction of huts, making beehives and household utensils (*Borketas*, chairs, and axe handles). Therefore, it is necessary to integrate it into the agroforestry systems and home-garden practices. *P. thonningii* is less known in its medicinal use as compared to in other African countries. Therefore, it is necessary to undergo more researches on its traditional medicinal value.

The results also showed that the status of *P. thonningii* is decreasing because of the pressures from various anthropogenic factors. The main threatening factors are agricultural expansion, firewood collection, harvesting for fencing, and overgrazing. Thus, there is a need to create public awareness and community-based management. Therefore, it is important to promote *P. thonningii* as a valuable tree to improve household food security, agricultural productivity, and income source of the local communities.

## Data Availability

All the data used to support this study have been deposited in the Dryad repository (Available at: 10.5061/dryad.9p8cz8wg3)

## Appendix

Plant species observed growing under/near *P. thonningii* tree

**Table:**

No.	Scientific name	Family	Local name (Benna Language)	Local name (Hamer Language	Habit
1	*Acacia robusta* Burch.	Fabaceae	Dhera	Dhera	T
2	*Acacia goetzei* Harms	Fabaceae	Arke	Arki	T
3	*Acacia hockii* De Wild.	Fabaceae	Chekanti	Chakanti	Sh
4	*Acacia seyal* Del.	Fabaceae	Pulanti	Fulante	T
5	*Acalypha fruticosa* Forssk.	Euphorbiaceae	Septi	Septi	Sh
6	*Adenium obesum* (Forssk.) Roem. & Schult.	Apocynaceae	Gurdo	Gurdo	Sh
7	*Becium obovatum* (E.Mey.ex Benth.	Lamiaceae	Buranbutso	Butamberohata	H
8	*Cissus rotundifolia* (Forssk.) Vahl	Vitaceae	Kelle	Kelle	C
9	*Clematis simensis* Fresen.	Ranunculaceae	Gayadenpo	Gayatenpo	C
10	*Cynodon dactylon* (L.) Pers.	Poaceae	Zersi	Zersi	G
11	*Dalbergia lactea* Vatke	Fabaceae	Zinzek	Zinzeke	Sh
12	*Entada abyssinica* Steud. Ex A. Rich.	Fabaceae	Longo	Longo	T
13	*Erythrococca abyssinica* Pax	Euphorbiaceae	-	Koda	Sh
14	*Euclea racemosa* Murr.	Ebenaceae	Unsi	Kunsi	Sh
15	*Gardenia ternifolia* Schumach. & Thonn.	Rubiaceae	Gembela	Gembela	T
16	*Grewia velutina* (Forssk.) Vahl.	Tiliaceae	Bereza	Berez	T
17	*Grewia villosa* Will.	Tiliaceae	Gergecha	Gergesho	Sh
18	*Hypoestes forskaoli*i (Vahl) R.Sch.	Acanthaceae	Busunta	Busente	H
19	*Ipomoea kituiensis* Vatke	Convolvulaceae	Gali	-	Sh
20	*Kalancboe citrina* Scweinfurth.	Crassulaceae	Gaya dempo	Gaya tampo	H
21	*Ozoroa insignis* Del.	Anacardiaceae	Selbena	Salbana	Sh
22	*Psychotria orophila* Petit	Rubiaceae	Shawbuno	Shawuno	Sh
23	*Pterolobium stellatum* (Forssk.) Brenan	Fabaceae	Beneka	Gorsa	C
24	*Rhus natalensis* Krauss	Anacardiaceae	Kufure	Kufuri	Sh
25	*Schoenoplectus subulatus* (Vahl) Lye	Cyperaceae	Kontsele	Kuntsele	H
26	*Solanum americanum* Mill.	Solanaceae	Tsepe	Tsepi	H
27	*Teclea nobilis* Del.	Rutaceae	Tsaki		Sh
28	*Terminalia brownii* Fresen.	Combretaceae	Ara	Ara	T
29	*Zanthoxylum chalybeum* Engl.	Rutaceae	Gedek	Gedeka	Sh
